# EMMY: The continued expansion of clinical applications of SGLT2 inhibitors

**DOI:** 10.21542/gcsp.2023.5

**Published:** 2023-01-30

**Authors:** Susy Kotit

**Affiliations:** 1 Aswan Heart Centre (AHC), Aswan, Egypt

## Abstract

**Introduction:** Myocardial infarction (MI) is a challenging clinical and public health problem and is a leading cause of morbidity and mortality worldwide. Heart failure (HF) is a common sequela of acute myocardial infarction (AMI), with an incidence of up to 40% among hospitalized patients and has important implications for treatment and prognosis. Sodium–glucose co-transporter 2 inhibitors (SGLT2i), such as empagliflozin, have been shown to reduce the risk of hospitalization and cardiovascular mortality in patients with symptomatic HF and have therefore been included in the European and American heart failure guidelines. However, trials investigating the effects of this drug class in patients following acute myocardial infarction are lacking.

**Study and Results:** The EMMY trial was conducted to assess the safety and efficacy of empagliflozin in patients with acute myocardial infarction (AMI). A total of 476 patients with AMI were randomly assigned to empagliflozin (10 mg) or matching placebo once daily within 72 h of percutaneous coronary intervention. The primary outcome was the N-terminal pro-hormone of brain natriuretic peptide (NT-proBNP) change over 26 weeks. Secondary outcomes included changes in echocardiographic parameters. NT-proBNP reduction was significantly greater in the empagliflozin group (−15% after adjusting for baseline NT-proBNP, gender, and diabetes status (*P* = 0.026)). Absolute left-ventricular ejection fraction improvement was 1.5% (*P* = 0.029) greater, mean E/e′ reduction was 6.8% (*P* = 0.015) greater, and left-ventricular end-systolic and end-diastolic volumes were lower by 7.5 mL (*P* = 0.0003) and 9.7 mL (*P* = 0.0015), respectively, in the empagliflozin group, compared with placebo. Seven patients were hospitalized for HF (3 in the empagliflozin group). Other predefined serious adverse events were rare and did not differ significantly between groups.

**Lessons learned:** The EMMY trial shows that early use of the SGLT2 inhibitor empagliflozin after acute myocardial infarction (MI) improves natriuretic peptide levels and markers of cardiac function and structure supporting the use of Empagliflozin in HF related to a recent MI.

## Introduction

Myocardial infarction (MI) is a challenging clinical and public health problem and is a leading cause of morbidity and mortality worldwide. Heart failure (HF) is a common sequela of acute myocardial infarction (AMI), with an incidence up to 40% among hospitalized patients^1,2^, and has important implications for treatment and prognosis^3–7^.

**Figure 1.**
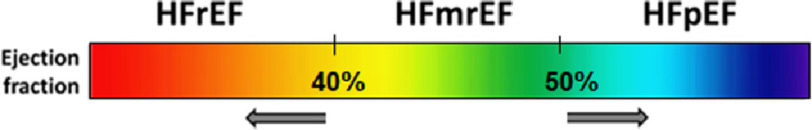
Classification of heart failure based on left ventricular ejection fraction (EF).

The primary classification of HF is based on left ventricular ejection fraction (LVEF) (Figure 1, Table 1)^8,9^. Serum N-terminal pro-brain natriuretic peptide (NT-proBNP) is routinely used as a diagnostic parameter of HF to predict outcomes, and to monitor the effects of therapy^10–14^.

**Table 1:** Classification of heart failure.

**TYPES OF HEART FAILURE**	
**Classification**	**Ejection Fraction (EF)**
Heart failure with reduced ejection fraction (HFrEF)	≤40%
Heart failure with mildly reduced ejection fraction (HFmrEF)	41–49%
Heart failure with preserved ejection fraction (HFpEF)	≥50%
Heart failure with improved ejection fraction (HFimpEF)	> 40%
(Baseline LVEF ≤40%, a ≥10-point increase from baseline LVEF, and a second measurement of LVEF > 40%)	

**Figure 2.**
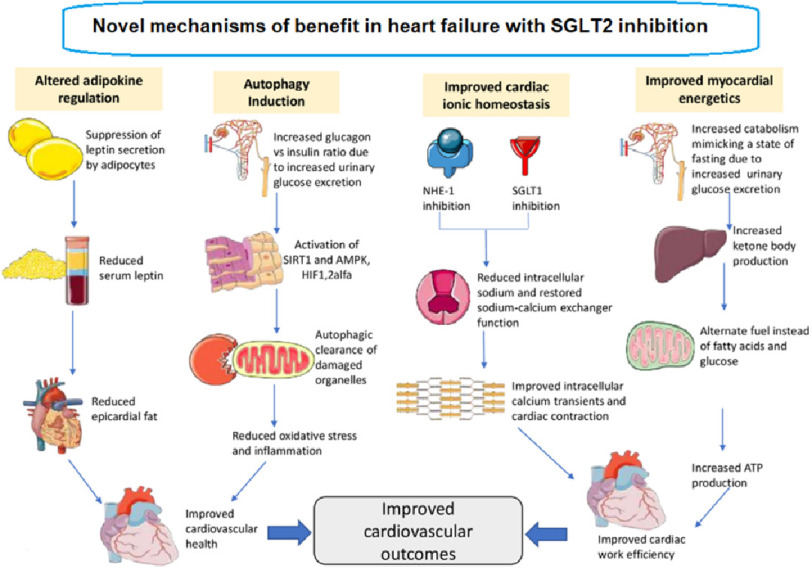
Schematic diagram showing proposed novel mechanisms of action of SGLT2 inhibitors in heart failure. AMPK, adenosine monophosphate-activated protein kinase; HIF, hypoxia-inducible factor; NHE, sodium-hydrogen exchanger; SGLT, sodium-glucose co-transporter; SIRT, sirtuin^24^.

**Figure 3.**
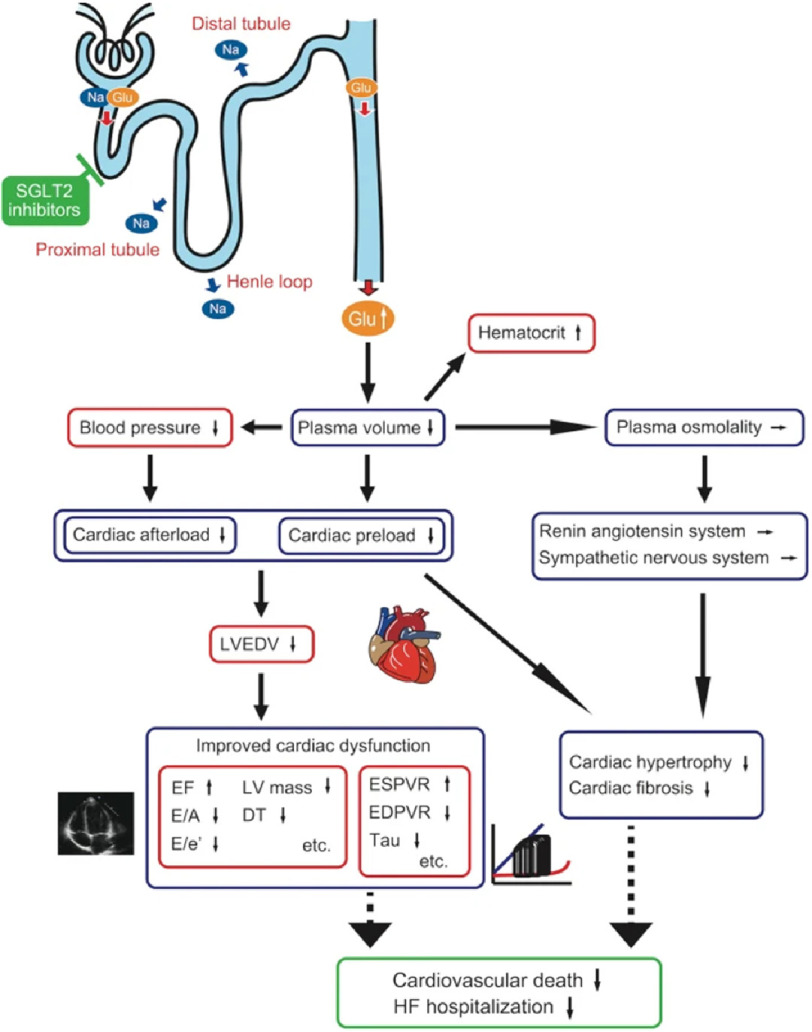
Effect of SGLT2 inhibitors on cardiac function and cardiovascular outcome. Osmotic diuresis mainly caused by urine glucose excretion leads to plasma volume reduction without activating renin angiotensin system and sympathetic nervous system. Plasma volume reduction leads to decreased cardiac workload resulting in the improvement of cardiac function and hence, favorable cardiovascular outcome. Blue box; functional and structural changes, Red box; clinical parameters, Green box; clinical outcome^25^.

Sodium–glucose co-transporter 2 inhibitors (SGLT2i) have been shown to reduce the risk of hospitalization for HF as well as all-cause mortality and cardiovascular mortality in chronic heart failure with reduced EF (HFrEF) (Figures 2, 3)^15–19^. The use of SGLT2i was therefore recently recommended in the European and American heart failure guidelines as part of first-line therapy for HFrEF^20,21^.

Empagliflozin, a SGLT2i, was the first drug shown in the EMPEROR-Preserved trial to improve the primary outcome of hospitalization for heart failure and cardiovascular death in HF patients with mildly reduced (HFmrEF) or preserved ejection fraction (HFpEF)^22^. The most recent American Heart Association (AHA)/American College of Cardiology (ACC)/Heart Failure Society of America (HFSA) guidelines also advocate SGLT2i use in patients with HFmrEF and HFpEF^21^.

In addition, in patients with obesity, who usually have higher rates of HF hospitalizations and worse health status, dapagliflozin has been shown to improve cardiovascular outcomes leading to greater symptom improvement with the additional benefit of causing modest weight loss^23^.

The favorable effects of SGLT2i on HF raise the question on the effect and safety of early start of empagliflozin following myocardial infarction (MI). However, until recently, no data was available for the use Empagliflozin in this patient population.

### The EMMY trial

The EMpagliflozin in patients with acute MYocardial infarction (EMMY) trial was conducted to assess the safety and efficacy of empagliflozin in patients with acute myocardial infarction (AMI)^26^.

EMMY was a prospective, multi-center, randomized, double-blind, placebo-controlled trial designed to evaluate the effect of empagliflozin 10 mg once daily (PO) initiated within 72 h after percutaneous coronary intervention (PCI) in addition to guideline-recommended post-MI therapy^27^ for 26 weeks on cardiac function and heart failure biomarkers (NT-proBNP) in patients with large acute MI, with or without diabetes, in comparison to a placebo, in 11 Austrian sites.

The primary outcome was a change in the NT-proBNP levels from randomization to week 26. Secondary endpoints included changes in LVEF, echocardiographic parameters for diastolic dysfunction, left-ventricular end-systolic (LVESV) and end-diastolic volume (LVEDV) at 6 and 26 weeks, and changes in ketone body, glycated haemoglobin concentrations and body weight. Additional endpoints were hospitalizations due to heart failure or other causes, duration of hospital stay and all-cause mortality. Key safety outcomes were the incidence of serious adverse events (SAEs), severe hypoglycaemic events, number of genital infections, number of ketoacidosis events, and acute liver or renal injury.

The patients included in the study were aged 18–80 years with a confirmed acute large MI (creatine kinase > 800 IU/L), high-sensitivity troponin T level or troponin I level (> 10-fold the upper limit of normal), and an estimated glomerular filtration rate > 45 mL/min/1.73 m2. Those with diabetes mellitus other than Type 2, a blood pH< 7.32, haemodynamic instability, acute symptomatic urinary tract infection or genital infection, an ongoing SGLT2i treatment or an SGLT2i treatment within 4 weeks prior to enrolment, were excluded.

A total of 476 patients were enrolled within 72 h after a PCI for acute MI and randomized in a 1:1 ratio to either oral empagliflozin 10 mg/day (n =237) or matching placebo once daily (n =239) via a Randomizer Software. Randomization was stratified by site, presence of Type 2 diabetes and by gender. Follow-up visits were scheduled at 6, 12, and 26 weeks.

The median age was 57 years (52–64), 18% were females and 13% had type 2 diabetes. Baseline characteristics were similar between treatment groups with a median baseline creatine kinase of 1673 (1202–2456) IU/L, troponin T of 3039 (2037–4856) ng/L, NT-proBNP of 1294 (757–2246) pg/mL, and median systolic blood pressure of 125 (117–131) mmHg.

Guideline-recommended post-MI medical therapy was initiated before randomization with >96% of patients receiving angiotensin converting enzyme inhibitor/angiotensin receptor blocker/angiotensin receptor–neprilysin inhibitor, beta-blocker and statins, and ∼40% receiving mineralocorticoid receptor antagonists.

Mean NT-proBNP levels declined in both groups during the study, but at week 12 and 26 the NT-proBNP level was 13% and 15% lower in the empagliflozin group, respectively, after adjustment for baseline NT-proBNP concentration, gender, and diabetes status (*P* = 0.026).

Left-ventricular systolic and diastolic function improved in both groups. Those in empagliflozin had a 1.7% (*p* = 0.014) and 1.5% (*p* = 0.029) higher LVEF in week 6 and 26, respectively. Left-ventricular diastolic function, as assessed by E/e′ ratio, was significantly improved, being 6.8% lower than placebo at 26 weeks (*P*  = 0.015). Echocardiographic parameters reflecting structural cardiac changes were significantly improved in the empagliflozin group, showing smaller LVESV (−7.5 mL, *p* = 0.0003) and LVEDV (−9.7 mL, *p* = 0.0015) values.

Ketone body (beta-hydroxybutyrate) concentrations showed a significantly greater increase in the empagliflozin group compared with placebo at 26 weeks (*p* = 0.11). Body weight decreased more in the empagliflozin group (−1.76 kg; *p* = 0.022). Within the small subgroup of participants with diabetes, there was no significant between-group difference in the degree of haemoglobin A1c lowering at week 26 (*p* = 0.11).

The number of genital infections did not differ significantly between the empagliflozin and placebo groups and no amputations, ketoacidosis, or severe hypoglycaemic episodes were reported throughout the follow up.

The median duration of hospital stay due to acute MI was 6.0 (3–9) days in both groups. There was a total of 72 adverse events with 63 hospitalizations, with no significant difference between the groups. Seven hospitalizations were due to heart failure (three in the empagliflozin group, four in placebo group).

There were three deaths during the study, all in the empagliflozin group, which were considered unrelated to treatment as the causes were large MIs, cardiogenic shock and lung cancer.

## Discussion

The results of this trial indicate that early administration of empagliflozin after AMI is superior to placebo in reducing NT-proBNP levels and improving markers of cardiac function and structure at 26 weeks.

These results strengthen the already known benefits of SGLT2 inhibitors in reducing the risk for HF hospitalization and cardiovascular death in patients with HFrEF as well as in high-risk groups, those with type 2 diabetes or chronic kidney disease. The trial supports the rapid expansion in indications for the use of SGLT2 inhibitors in a wide spectrum of HF (EMPEROR-Preserved^28,29^, EMPEROR-Reduced^30,31^, DAPA-HF^32–34^.

The EMMY trial assessed the benefit of empagliflozin on surrogate markers of HF after MI. However, the results of the EMMY trail are based on a relatively homogenous, low-risk population, without HF symptoms or cardiac dysfunction (median baseline EF of almost 50% and estimated glomerular filtration rate (eGFR) of more than 45 mL/min per 1.73 m2 (median, 90 mg/min per 1.73 m2), and does not provide expectations and benefits in a higher risk patient population. The results are further limited by the fact that only 18% of participants were women and the majority of the participants did not have type 2 diabetes mellitus.

The clinical outcomes remain therefore unclear as the sample is too small and the follow-up too short to assess potential differences in actual clinical events between groups. Regardless of the limitations, the EMMY trial represents an important step forward for SGLT2 inhibitors in the management of post-MI heart failure and increases the optimism that these drugs may provide clinical benefit to the post-MI population.

Larger trials should be performed in order to assess and fully understand the safety and efficacy of SGLT2 inhibitors after MI. To that end, the ongoing large  DAPA-MI^35^ and EMPACT-MI^36^ trials are both enrolling MI survivors with new cardiac dysfunction to test SGLT2 inhibition in this setting. Further insights will be provided by these trials, as they will test whether dapagliflozin and empagliflozin, respectively, can lower the risk for HF hospitalization and death in patients with new cardiac dysfunction after MI. EMPACT-MI will additionally evaluate patients with preserved EF with or at high risk of new onset HF in the setting of MI.

Both trials are enrolling patients with an eGFR down to 20 mL/min per 1.73 m2, enabling a broad evaluation of the possible renal safety and efficacy of SGLT2 inhibitors. The trials are expected to be completed in 2023.

### Lessons learned

The EMMY trial shows that early use of the SGLT2 inhibitor empagliflozin after acute myocardial infarction (MI) improves natriuretic peptide levels and markers of cardiac function and structure supporting the use of empagliflozin in HF related to a recent MI.

The trial showed that compared with a placebo, empagliflozin led to a significant decline in mean NT-proBNP levels, improvement of left-ventricular systolic and diastolic function and echocardiographic parameters reflecting structural cardiac changes. However, empagliflozin does not seem to have an impact on adverse events, duration of hospital stay or the number of HF hospitalizations.

The EMMY trial represents an important step forward for SGLT2 inhibitors in the management of post-MI heart failure and supports the rapid expansion in indications for the use of SGLT2 inhibitors in a wide spectrum of HF. Larger trials are underway to assess and fully understand the safety and efficacy of SGLT2 inhibitors after MI.
